# News and Coming Events

**Published:** 1909-03-20

**Authors:** 


### News and Coming Events.

The Prince and Princess of Wales on March 10 paid a visit to the Royal College of Surgeons, in Lincoln's Inn Fields. Their Royal Highnesses were attended by Sir Frederick Treves, and were received at the College by the President, Mr. Henry Morris; the Conservator, Professor Arthur Keith; and the Secretary, Mr. S. Forrest Cowell. After being conducted over the Hunterian Museum their Royal Highnesses proceeded to the Council Chamber, where a special collection of specimens was displayed.

Lord Northcote will preside at the annual festival dinner at King's College Hospital, at the Hotel Cecil on Monday, June 21. The Committee for the removal of the Hospital to South London has received a further donation of £1,000 from Messrs. W. H. Smith and Son in aid of the fund; and £2,500 from the trustees of the late Mr. Zunz, this being a second instalment of a grant of £10,000 promised to the fund on the condition that a ward in the new hospital, containing not less than twenty beds, is to be called the "Annie Zunz Ward" in perpetuity.

The Annual Court of Governors of the Seamen's Hospital Society was held on March 11 under the presidency of Mr. Perceval A. Nairne, Chairman of the Committee. The report shows that a larger number of patients have been treated during 1908 than in any previous year, but that the income was not equal to the expenditure by nearly £3,000. Two-thirds of this deficit, however, was due to expenditure on structural alterations and improvements. Sir Walter Hunt-Grubbe, Sir Stephen Mackenzie, Dr. T. L. Rogers, and Dr. Thomas Seccombe, R.N., were elected Vice-Presidents, and Mr. E. Lydekker, Capt. E. B. Pusey, R.N., General the Hon. Sir Reginald Talbot, K.C.B., and Mr. John T. Wimble, members of the Committee of Management. The report urged the public to increase their support of the *Dreadnought*, which by its attention to sick and injured seamen carries on a work of inestimable advantage to the community.

At the last meeting of the Council of the Royal College of Surgeons of England on March 11, Dr. Nicholas Weliaminoff, Privy Councillor of State and Professor of Surgery at the Imperial Military Academy of Medicine, St. Petersburg, having been unanimously elected an Honorary Fellow of the College, was presented to the Council and signed the roll. Dr. Weliaminoff was nominated for election in 1900, when the Honorary Fellowship was instituted, but was unable to come to England at that time in order to be admitted. Sir Gilbert Blane Medals have been awarded to Staff Surgeons C. R. Nicholson, *H.M.S. Egmont*, 1906-7, and A. W. B. Livesay, M.B., *H.M.S. Bonaventure*, 1907. A report from the Committee appointed to prepare a new by-law relating to the admission of women to examination for the diplomas of the College was received and considered, and the proposed new by-law will come before the Council at their next meeting, for further consideration and final approval. The attention of the Council was called to the petition of the British Medical Association for the grant of a Charter of Incorporation. A committee was appointed to consider the terms of the proposed charter and its bearing upon the position of the College and the rights of its Fellows and members. Mr. Henry Morris was re-elected the representative of the College in the General Medical Council for the next five years, and Mr. F. Richardson Cross was appointed the Bradshaw Lecturer for the ensuing collegiate year.

The annual general meeting of the Poplar Hospital for Accidents, East India Road, Poplar, E., will be held at the hospital on Tuesday, March 23, at 4 o'clock P.M.

The thirty-first ordinary general meeting of the Home Hospitals Association for Paying Patients will be held at Fitzroy House, 16, 17, and 18 Fitzroy Square, London, W., on Friday, March 26, 1909, at 4 P.M., his Grace the Duke of Northumberland, K.G., in the chair.

The National League for Physical Education and Improvement will hold a meeting at 137 Harley Street, W., by permission of Mrs. Bickerton, on Wednesday, March 24, at 3.30 P.M. Mrs. Carl Meyer will take the chair, and an address will be delivered by F. E. Fremantle, Esq., F.R.C.S., D.P.H., county medical officer and school medical officer for Herts.

A Reuter's telegram from Cairo says: With reference to the statement that in two cases of smallpox among the employés of a Lancashire cotton mill the infection had been traced by the medical authorities to cotton imported from Egypt, it is officially stated that, while there are some cases of smallpox here, the number is nothing unusual and there is no epidemic. The actual state of the general health of Egypt is not only excellent but is unusually so.

The Under Secretary for Foreign Affairs, in reply to a question in the House of Commons on March 15, stated that in the year 1907 the death rate per thousand in England and Wales was 15, in France 20.2, in the German Empire 18.9, in London 14.6, in Paris 18.5, and in Berlin 15.4. The percentage of infants who died within a year of their birth during the year 1907 was in England and Wales 11.8, in the German Empire 17.6, in France (in 1906) 14.3; in London 11.6, in Paris 10.5, and in Berlin 16.3.

The Lord Mayor presided at the Eighty-first Annual Meeting of the Royal Free Hospital which was held at the Mansion House on March 10. Amongst the speakers was Dr. Walter Carr, who in an eloquent speech defended the ever increasing expenditure which a large general hospital must inevitably incur, and emphasised the urgent need of the hospital for a new out-patient department, pointing out that the minor ailments of life are responsible for a vast amount of suffering, distress, and unemployment. Moreover in the out-patient department the early beginnings of serious organic diseases are recognised at a period when they are still capable of being successfully treated; the cases are sorted out and the students obtain some of their most important training.

The annual meeting of the Governors of the Central London Ophthalmic Hospital, Gray's Inn Road, was held on March 11, under the chairmanship of Mr. C. H. R. Wollaston. The report showed that the out-patients during the year numbered 12,104, and the in-patients 408; while 546 accidents and urgent cases were also attended to. The ordinary income amounted to £1,799, and the ordinary expenditure was £1,952. The chief concern of the committee at present is the urgent need of rebuilding, and a suitable site has been secured in the neighbourhood. Plans have been prepared, and it is proposed to commence the work forthwith; £10,000 has been subscribed, but £15,000 more is needed. The lease of the existing building has nearly expired.

